# *Mir125b-2* imprinted in human but not mouse brain regulates hippocampal function and circuit in mice

**DOI:** 10.1038/s42003-023-04655-y

**Published:** 2023-03-14

**Authors:** Ming-Yi Chou, Xuhui Cao, Kuan-Chu Hou, Meng-Han Tsai, Chih-Yu Lee, Meng-Fai Kuo, Vin-Cent Wu, Hsin-Yi Huang, Schahram Akbarian, Sheng-Kai Chang, Chung-Yi Hu, Shu-Wha Lin, Hsien-Sung Huang

**Affiliations:** 1 Graduate Institute of Brain and Mind Sciences, College of Medicine, National Taiwan University, Taipei, 10051 Taiwan; 2 Graduate Institute of Medical Genomics and Proteomics, College of Medicine, National Taiwan University, Taipei, 10051 Taiwan; 3 Division of Neurosurgery, Department of Surgery, National Taiwan University Hospital and National Taiwan University College of Medicine, Taipei, 10051 Taiwan; 4 Department of Internal Medicine, National Taiwan University Hospital and College of Medicine, National Taiwan University, Taipei, 10051 Taiwan; 5 Department of Pathology, National Taiwan University Hospital and College of Medicine, National Taiwan University, Taipei, 10051 Taiwan; 6 Department of Psychiatry, Icahn School of Medicine at Mount Sinai, New York, NY 10029 USA; 7 Department of Clinical Laboratory Sciences and Medical Biotechnology, College of Medicine, National Taiwan University, Taipei, 10051 Taiwan

**Keywords:** Epigenetics in the nervous system, Development of the nervous system, Diseases of the nervous system, miRNAs, Synaptic transmission

## Abstract

Genomic imprinting predominantly occurs in the placenta and brain. Few imprinted microRNAs have been identified in the brain, and their functional roles in the brain are not clear. Here we show paternal, but not maternal, expression of *MIR125B2* in human but not mouse brain. Moreover, *Mir125b-2*^*m*^^−^^*/p*^^−^ mice showed impaired learning and memory, and anxiety, whose functions were hippocampus-dependent. Hippocampal granule cells from *Mir125b-2*^*m*^^−^^*/p*^^−^ mice displayed increased neuronal excitability, increased excitatory synaptic transmission, and decreased inhibitory synaptic transmission. Glutamate ionotropic receptor NMDA type subunit 2A (*Grin2a*), a key regulator of synaptic plasticity, was physically bound by miR-125b-2 and upregulated in the hippocampus of *Mir125b-2*^*m*^^−^^*/p*^^−^ mice. Taken together, our findings demonstrate *MIR125B2* imprinted in human but not mouse brain, mediated learning, memory, and anxiety, regulated excitability and synaptic transmission in hippocampal granule cells, and affected hippocampal expression of *Grin2a*. Our work provides functional mechanisms of a species-specific imprinted microRNA in the brain.

## Abstract (web summary)

Knockout of *Mir125b-2* in mice leads to defects in hippocampus-related behaviors and affects hippocampal expression of *Grin2a* and NMDAR-mediated currents.

## Introduction

Genomic imprinting displaying parent-of-origin-specific gene expression is a special case of epigenetic mechanisms^1,2^. Genomic imprinting predominantly occurs in the placenta and brain^1,3–8^. *MicroRNAs (miRNAs)* are an extensive class of small non-coding RNAs that repress gene expression post-transcriptionally through transcript destabilization or translational inhibition^9^. Comparative genomics studies show that primate-specific miRNAs contribute considerably to human-specific miRNAs^10^. miRNAs are highly expressed in neurons where they play significant roles during neuronal differentiation, synaptogenesis, and plasticity^11^. Moreover, accumulating evidence strongly suggests that the dysfunction of miRNAs contributes to various neurological and psychiatric disorders^12^. To date, few imprinted miRNAs have been identified in the brain^4,5,13^, and their functional roles in the brain are not clear.

miR-125 is a miRNA that is highly conserved from nematodes to humans and consists of three homologs: miR-125a, miR-125b-1, and miR-125b-2^14^. miR-125b-2, an orthologue of lin-4, was first identified in *Caenorhabditis elegans* (*C. elegans*), which regulates developmental timing^15^. Moreover, miR-125b-2 is one of the most abundant miRNAs in the mouse and human brain^16,17^. In addition, miR-125b-2 regulates neuronal differentiation^18–20^, dendritic spine morphology^21^, and synaptic maturation^21^. Importantly, aberrant expression of miR-125b-2 occurs in several brain disorders such as Down syndrome^22^, acute ischemic stroke^23^, and Alzheimer’s disease^24,25^. However, it is unclear whether *Mir125b-2* is imprinted in the brain and how miR-125b-2 contributes to brain function and related brain disorders.

To investigate the imprinting status of *Mir125b-2* in the brain, we investigated human brains in cohorts of family trios or quartets and found *MIR125B2* was paternally, but not maternally, expressed in human brains. Imprinting profiling of *Mir125b-2* was further continued with mouse brain by generating *Mir125b-2* knockout mice. We analyzed levels of miR-125b-2 in the cerebral cortex from paternal (*Mir125b-2*^*m+/p*^^−^) and maternal (*Mir125b-2*^*m*^^−^^*/p+*^) *Mir125b-2* knockout mice and their corresponding control wild-type mice and found *Mir125b-2* was biallelically expressed in the cerebral cortex.

Next, to investigate the functional roles of miR-125b-2 in the brain, *Mir125b-2* homozygous (*Mir125b-2*^*m*^^−^^*/p*^^−^) knockout mice and their corresponding control wild-type mice were generated. We found deficits of hippocampus-related behaviors such as learning and memory and anxiety in *Mir125b-2*^*m*^^−^^*/p*^^−^ mice compared with their controls. Moreover, the hippocampus of *Mir125b-2*^*m*^^−^^*/p*^^−^ mice showed increased neuronal excitability and excitatory synaptic transmission and decreased inhibitory synaptic transmission. Furthermore, glutamate ionotropic receptor NMDA type subunit 2A (*Grin2a*), a regulator of synaptic plasticity underlying learning and memory, was physically bound by miR-125b-2 and upregulated in the hippocampus of *Mir125b-2*^*m*^^−^^*/p*^^−^ mice compared with their controls. The peak of NMDA receptor (NMDAR)-mediated currents was increased in the hippocampus of *Mir125b-2*^*m*^^−^^*/p*^^−^ mice. Collectively, our findings demonstrated that *Mir125b-2* is imprinted in human but not mouse brain, regulates hippocampal circuit and function, and affects the hippocampal expression of *Grin2a* and NMDAR-mediated currents.

## Results

### *MIR125B2* is imprinted in the human brain and adrenal gland

**Fig. 1:**
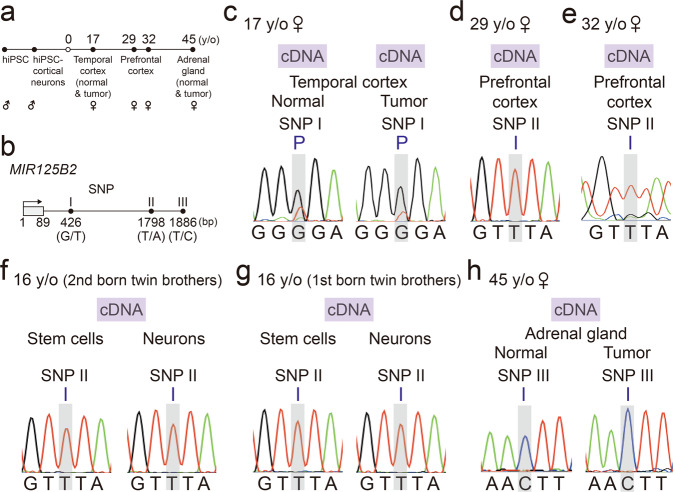
*MIR125B2* is imprinted in the human brain and adrenal gland. **a** Schematic of human induced pluripotent stem cells (hiPSCs), hiPSCs-derived cortical neurons, tissues, disease status of tissues, and corresponding gender and age of hiPSCs and tissues in this study. **b** Schematic of *MIR125B2* genomic locus and all SNP sites used in this study. Arrow indicates the direction of transcription; the number indicates nucleotide positions relative to the transcription start site of *MIR125B2*. Lengths of sequences between SNP sites are not drawn to scale. **c** Parent-of-origin-specific allelic expression of *MIR125B2* in normal and tumor tissue from the temporal cortex of a 17-year-old female was determined by Sanger sequencing. **d** Parent-of-origin-specific allelic expression of *MIR125B2* in the prefrontal cortex of a 29-year-old female was determined by Sanger sequencing. **e** Parent-of-origin-specific allelic expression of *MIR125B2* in the prefrontal cortex of a 32-year-old female was determined by Sanger sequencing. Parent-of-origin-specific allelic expression of *MIR125B2* in the human induced pluripotent stem cells (hiPSCs) and hiPSCs-derived cortical neurons from 16-year-old twin brothers (**f**) and (**g**). **h** Parent-of-origin-specific allelic expression of *MIR125B2* in the adrenal gland from a 45-year-old female (normal and tumor tissue) was determined by Sanger sequencing. “P” = paternal expression. “I” = imprinting expression. Single nucleotide polymorphism site is indicated by the gray column.

To examine the imprinting status of *MIR125B2* in the human brain, we performed Sanger sequencing on human blood genomic DNA and brain cDNA obtained from human family trios and quartets. Tissue included available human postmortem and surgical resection brains of different ages and disease statuses and human induced pluripotent stem cells (hiPSCs)-derived cortical neurons (Fig. 1a). Distinguishable single nucleotide polymorphisms (SNPs) (Fig. 1b, Supplementary Fig. 1) were identified from our available human cohorts. Analysis showed *MIR125B2* was paternally, but not maternally, expressed in the temporal cortex of normal and tumor tissue of a 17-year-old female (Fig. 1c). Furthermore, *MIR125B2* was imprinted in the prefrontal cortex of 29-, and 32-year-old females (Fig. 1d, e), and hiPSCs and hiPSCs-derived cortical neurons of 16-year-old twin brothers (Fig. 1f, g). To test whether or not *MIR125B2* was imprinted in the human brain exclusively, the imprinting status of *MIR125B2* was examined in the adrenal gland from normal and tumor tissue of a 45-year-old female and found *MIR125B2* was also imprinted in the human adrenal gland. Collectively, our results showed *MIR125B2* is imprinted in the human brain and adrenal gland.

### *Mir125b-2* is not imprinted in the mouse brain, but is highly expressed in neonatal brains and regulates brain size

**Fig. 2:**
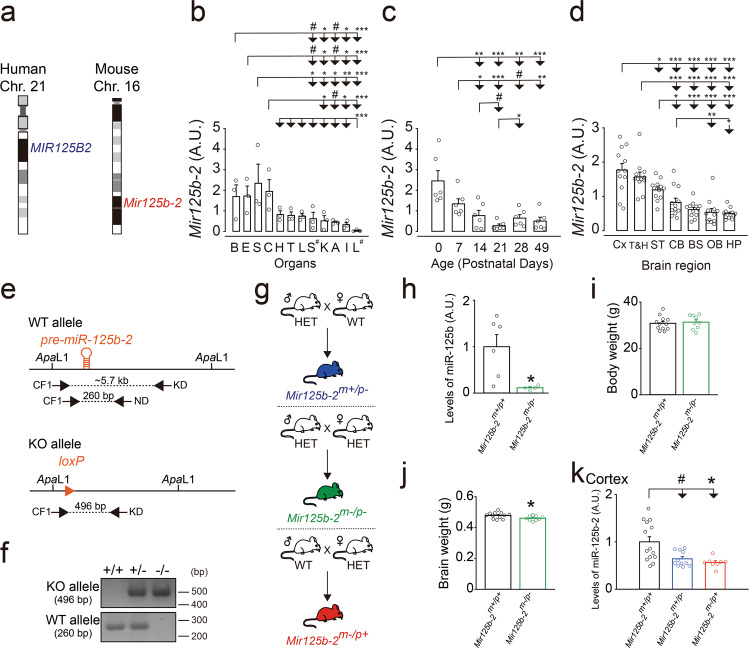
Expression profiling of *Mir125b-2* and validation of *Mir125b-2* knockout mice. **a** Schematic diagram of chromosome locations of human *MIR125b2* and mouse *Mir125b-2*. Levels of *Mir125b-2* were measured in male postnatal day 28 (P28) mice with qPCR in various organs (**b**, *n* = 3), from the whole brain at distinct developmental stages (**c**, *n* = 9), and in multiple regions of the brain (**d**, *n* = 12). One-way ANOVA with Fisher LSD method *post hoc* comparison. **e** Schematic diagram of targeting locus of *pre-miR-125b-2* in WT mice (top) and *Mir125b-2* knockout mice (bottom). The bottom schematic indicates the *pre-miR-125b-2* sequence was completely deleted. **f** *Mir125b-2* knockout mice were genotyped by PCR with WT and KO allele primers shown in (**e**). **g** Schematic diagram of mouse mating strategy for generating paternal *Mir125b-2* knockout (*Mir125b-2*^*m+/p*^^−^), maternal *Mir125b-2* knockout (*Mir125b-2*^*m*^^−^^*/p+*^), and homozygous *Mir125b-2* knockout (*Mir125b-2*^*m*^^−^^*/p*^^−^) offspring. WT = wild-type mice; HET = heterozygous knockout mice. **h** Expression levels of mature miR-125b-2 were measured with qPCR from the hippocampus of adult *Mir125b-2*^*m*^^−^^*/p*^^−^ mice and their corresponding wild-type (*Mir125b-2*^*m+/p+*^) controls. Mann–Whitney rank-sum test. *Mir125b-2*^*m+/p+*^, *n* = 6 mice; *Mir125b-2*^*m*^^−^^*/p*^^−^, *n* = 4 mice. **i** Body weight and **j** brain weight were measured from male adult *Mir125b-2*^*m*^^−^^*/p*^^−^ mice and their corresponding wild-type (*Mir125b-2*^*m+/p+*^) controls. Student’s *t*-test, two-tailed. *Mir125b-2*^*m+/p+*^, *n* = 12 mice; *Mir125b-2*^*m*^^−^^*/p*^^−^, *n* = 9 mice. Expression levels of *Mir125b-2* were measured in the cerebral cortex at P28 with qPCR (**k**) from *Mir125b-2*^*m+/p*^^−^ mice (blue bar), *Mir125b-2*^*m*^^−^^*/p+*^ mice (red bar), and corresponding controls (black bar). *n* = 11 mice for *Mir125b-2*^*m+/p*^^−^ mice; *n* = 8 for *Mir125b-2*^*m*^^−^^*/p+*^ mice; *n* = 14 for controls. Mann–Whitney rank-sum test, ^#^*P* < 0.1, *P < 0.05, **P < 0.01, ***P < 0.001. All data are presented as mean ± s.e.m. B: brain. E: eye. S: spleen. C: colon. H: heart. T: testis. L: lung. S^#^: stomach. K: kidney. A: adrenal gland. I: small intestine. L^#^: liver. Cx: cerebral cortex. T&H: thalamus and hypothalamus. ST: striatum. CB: cerebellum. BS: brainstem. OB: olfactory bulb. HP: hippocampus.

The location of human *MIR125B2* was on the smallest human autosome, chromosome 21 (Fig. 2a, left). However, mouse *Mir125b-2* was located on chromosome 16, which is an average-sized chromosome in the mouse (Fig. 2a, right). Because the imprinting status is regulated by the imprinting control center, we wondered whether the imprinting of human *MIR125B2* was conserved in mouse *Mir125b-2*. However, prior to investigating the imprinting status of *Mir125b-2* in the mouse brain, we first profiled the expression patterns of mouse miR-125b-2 in various organs, distinct developmental stages of the brain, and diverse brain regions and found that miR-125b-2 was indeed enriched in mouse brain (Fig. 2b), which is consistent with previous findings^16^. Moreover, expression of miR-125b-2 reached a maximum at postnatal day 0 (P0) and dramatically decreased during mouse brain development (Fig. 2c). Examination of diverse regions of the mouse brain at postnatal day 28 (P28) showed the greatest levels of expression of miR-125b-2 were in the cortex, striatum, thalamus, and hypothalamus (Fig. 2d).

Next, we generated *Mir125b-2* conventional knockout (KO) mice (Fig. 2e), and their genotypes were verified by PCR with corresponding KO and WT primers (Fig. 2f). In addition, their expression levels were confirmed by qPCR (Fig. 2h). Although *Mir125b-2* homozygous knockout (*Mir125b-2*^*m*^^−^^*/p*^^−^) mice displayed normal body weight compared with WT controls (Fig. 2i), brain weight was lower (Fig. 2j). To further investigate what contributes to the decreased brain weight in *Mir125b-2*^*m*^^−^^*/p*^^−^ mice, we examined protein markers for the integrity of neurons (NF-200 and NeuN), glia (GFAP), excitatory synapses (PSD95 and SYN1), and inhibitory synapses (Gephyrin) in the hippocampus (Supplementary Fig. 2) and cerebral cortex (Supplementary Fig. 3) of *Mir125b-2*^*m*^^−^^*/p*^^−^ mice and their corresponding controls using western blot analysis. No differences were observed for any of the above protein markers in either the cerebral cortex or the hippocampus. Next, to investigate the imprinting status of *Mir125b-2* in the mouse brain, paternal (*Mir125b*^*m+/p*^^−^) and maternal (*Mir125b-2*^*m*^^−^^*/p+*^) *Mir125b-2* knockout mice and their corresponding control wild-type (*Mir125b*^*m+/p+*^) mice were generated (Fig. 2g). When levels of miR-125b-2 were measured in the cerebral cortex from *Mir125b*^*m+/p*^^−^ mice, *Mir125b-2*^*m*^^−^^*/p+*^ mice, and their corresponding control wild-type (*Mir125b*^*m+/p+*^) mice, we found *Mir125b-2* was biallelically expressed in the cerebral cortex (Fig. 2k). In summary, *Mir125b-2* was highly expressed in neonatal brains, affected brain size, and was not imprinted in mouse brain.

### *Mir125b-2*^*m*^^−^^*/p*^^−^ mice displayed impaired hippocampus-related behaviors such as learning and memory and anxiety

**Fig. 3:**
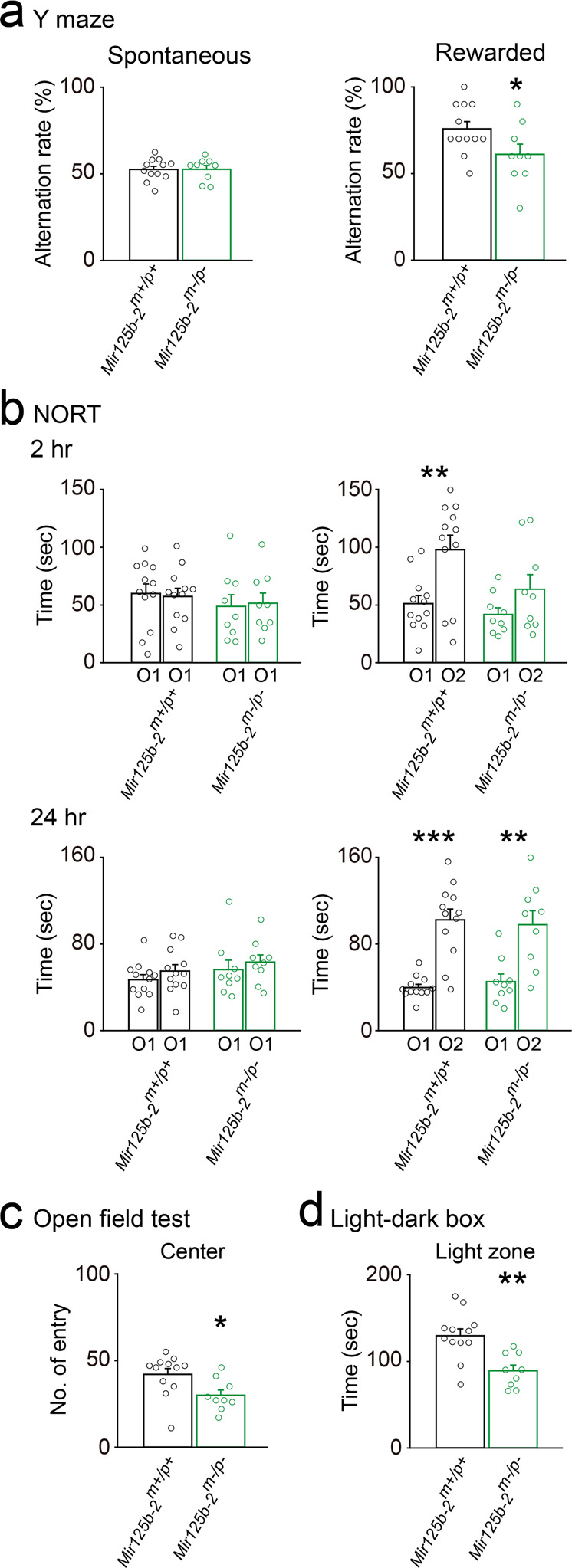
*Mir125b-2*^*m*^^−^^*/p*^^−^ mice showed impaired hippocampus-related behaviors. Bar graphs showing differences in behavioral tests from *Mir125b-2*^*m*^^−^^*/p*^^−^ mice and their corresponding wild-type controls (*Mir125b-2*^*m+/p+*^). **a** Alternation rate (%) of the Y-maze test: spontaneous (left) and rewarded (right). **b** Results of novel object recognition test (NORT). Training phase (left panels): time spent with the same two objects (O1) for 2 h (top) and 24 h (bottom). Test phase (right panels): time spent with two different objects (O1 and O2) for 2 h (top) and 24 h (bottom). **c** Bar graph showing the number of entries into the center of the field for *Mir125b-2*^*m+/p+*^ and *Mir125b-2*^*m*^^−^^*/p*^^−^ mice during the open field test. **d** Bar graph showing time spent in the light zone for *Mir125b-2*^*m+/p+*^ and *Mir125b-2*^*m*^^−^^*/p*^^−^ mice during the light-dark box test. Student’s *t*-test, two-tailed. *P < 0.05, **P < 0.01, ***P < 0.001, ****P < 0.0001. *Mir125b-2*^*m+/p+*^, *n* = 12 mice; *Mir125b-2*^*m*^^−^^*/p*^^−^, *n* = 9 mice. All data are the mean ± s.e.m.

miR-125b-2 regulates neuronal differentiation^18–20^, dendritic spine morphology^21^, and synaptic maturation^21^. However, the in vivo functions of miR-125b-2 in the brain have not been established. To address this issue, we focused on hippocampus-related behaviors in *Mir125b-2*^*m*^^−^^*/p*^^−^ mice and their controls because aberrant expression of miR-125b-2 has been identified in persons with cognition-related dysfunction^22–25^. First, spatial short-term working memory behaviors were evaluated with the rewarded Y-maze test, which showed *Mir125b-2*^*m*^^−^^*/p*^^−^ mice displayed decreased rewarded alternation rates compared with their controls (Fig. 3a, right) and performed normally for spontaneous alternation rates compared with their controls (Fig. 3a, left). Next, short-term (2 h) and long-term (24 h) memory was evaluated with the novel object recognition test (NORT). We found *Mir125b-2*^*m*^^−^^*/p*^^−^ mice displayed deficits of short-term (Fig. 3b, top) but not long-term (Fig. 3b, bottom) memory. This data was consistent with results from the rewarded Y-maze test (Fig. 3a, right). We also evaluated fear-related learning and memory using the fear conditioning test and did not find any deficit in *Mir125b-2*^*m*^^−^^*/p*^^−^ mice compared with their control mice (Supplementary Fig. 4a).

The hippocampus not only plays distinct roles in learning and memory but also in anxiety^26^. Evaluation of anxiety in *Mir125b-2*^*m*^^−^^*/p*^^−^ mice compared with controls was conducted with the open field test and light-dark box test. *Mir125b-2*^*m*^^−^^*/p*^^−^ mice made fewer entries into the center of the field in the open field test (Fig. 3c) and spent less time in the light zone of the light-dark box test (Fig. 3d). Both of these results suggested *Mir125b-2*^*m*^^−^^*/p*^^−^ mice experience more anxiety compared with their controls. Anxiety-like behavior was further evaluated with the elevated plus maze test (Supplementary Fig. 4b). Moreover, depressive-like behavior was examined with the forced swim test (Supplementary Fig. 4c); no differences were observed between *Mir125b-2*^*m*^^−^^*/p*^^−^ and control mice (Supplementary Fig. 4b, c).

The presence of motor-related dysfunctions can be a confounding variable for behavioral tests. Thus, the open field test, rotarod test, and catwalk test were conducted to eliminate the possibility of dysfunctional motor behaviors. *Mir125b-2*^*m*^^−^^*/p*^^−^ mice displayed hypoactivity but normal motor speed, coordination, and balance (Supplementary Fig. 4d–f). Together, these data indicated *Mir125b*^*m*^^−^^*/p*^^−^ mice showed deficits in hippocampus-related behaviors such as learning and memory and anxiety.

### Hippocampal granule cells in *Mir125b-2*^*m*^^−^^*/p*^^−^ mice demonstrated increased neuronal excitability

**Fig. 4:**
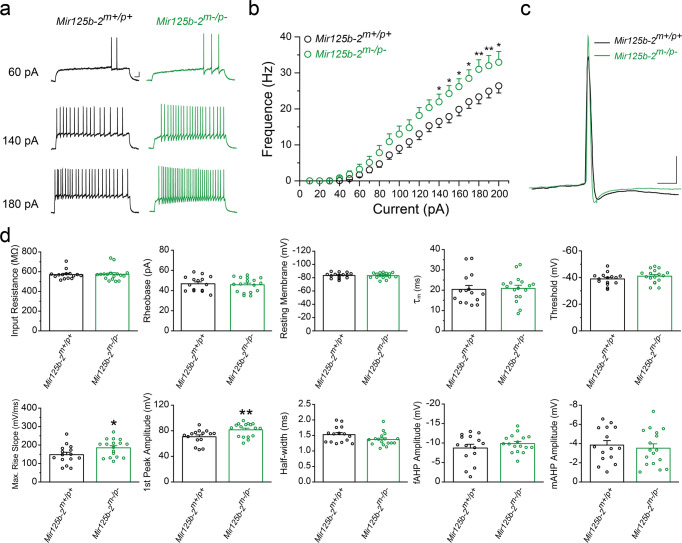
Hippocampal granule cells of *Mir125b-2*^*m*^^−^^*/p*^^−^ mice displayed increased neuronal excitability. **a** Representative responses of hippocampal granule cells from wild-type (*Mir125b-2*^*m+/p+*^) and *Mir125b-2* homozygous knockout (*Mir125b-2*^*m*^^−^^*/p*^^−^) mice to different current injections. Scale bar represents 10 mV and 100 ms. **b** Quantification of the frequency of action potential firing during the indicated magnitude of current injection. (*Mir125b-2*^*m+/p+*^, *n* = 15 slices from six female mice; *Mir125b-2*^*m*^^−^^*/p*^^−^, *n* = 17 slices from seven female mice). **c** Representative traces of recordings of first action potential from hippocampal granule cells of *Mir125b-2*^*m+/p+*^ and *Mir125b-2*^*m*^^−^^*/p*^^−^ mice. Scale bar represents 10 mV and 100 ms. **d** Bar graphs show the input resistance, rheobase, resting membrane potential, membrane time constant from voltage response between 0 pA and 1-s hyperpolarizing current injection of −30 pA, and threshold potential (top); the maximum rise slope of first action potentials, the first action potential amplitudes, half-width, and amplitudes of fast and medium after-hyperpolarization potential (fAHP and mAHP) (bottom). Statistical significance was assessed using two-way repeated measured ANOVA with Holm–Sidak *post hoc* comparison (**b**), or Student’s *t*-test, two-tailed (**d**), *p < 0.05, **p < 0.01, ***p < 0.001. The significance for (**d**) disappeared after Bonferroni correction. All data are the mean ± s.e.m.

To investigate neuronal mechanisms for deficits in hippocampus-related behaviors from *Mir125b-2*^*m*^^−^^*/p*^^−^ mice (Fig. 3), we measured the neuronal excitability of hippocampal granule cells from *Mir125b-2*^*m*^^−^^*/p*^^−^ mice. The evoked neuronal firing activity was evaluated by injection of depolarizing current steps (Fig. 4a). Compared with wild-type (*Mir125b-2*^*m+/p+*^) mice, the mean evoked firing rate of *Mir125b-2*^*m*^^−^^*/p*^^−^ mice increased at 140 pA current step (Fig. 4b), and the frequency also significantly increased after the depolarization current step. However, there was no significant difference in intrinsic properties between *Mir125b-2*^*m+/p+*^ and *Mir125b-2*^*m*^^−^^*/p*^^−^ mice after Bonferroni correction (Fig. 4c–d). Our results showed that loss of *Mir125b-2* increased the neuronal excitability of hippocampal granule cells.

### Excitatory synaptic transmission increased, and inhibitory synaptic transmission decreased in the hippocampal dentate gyrus of *Mir125b-2*^*m*^^−^^*/p*^^−^ mice

**Fig. 5:**
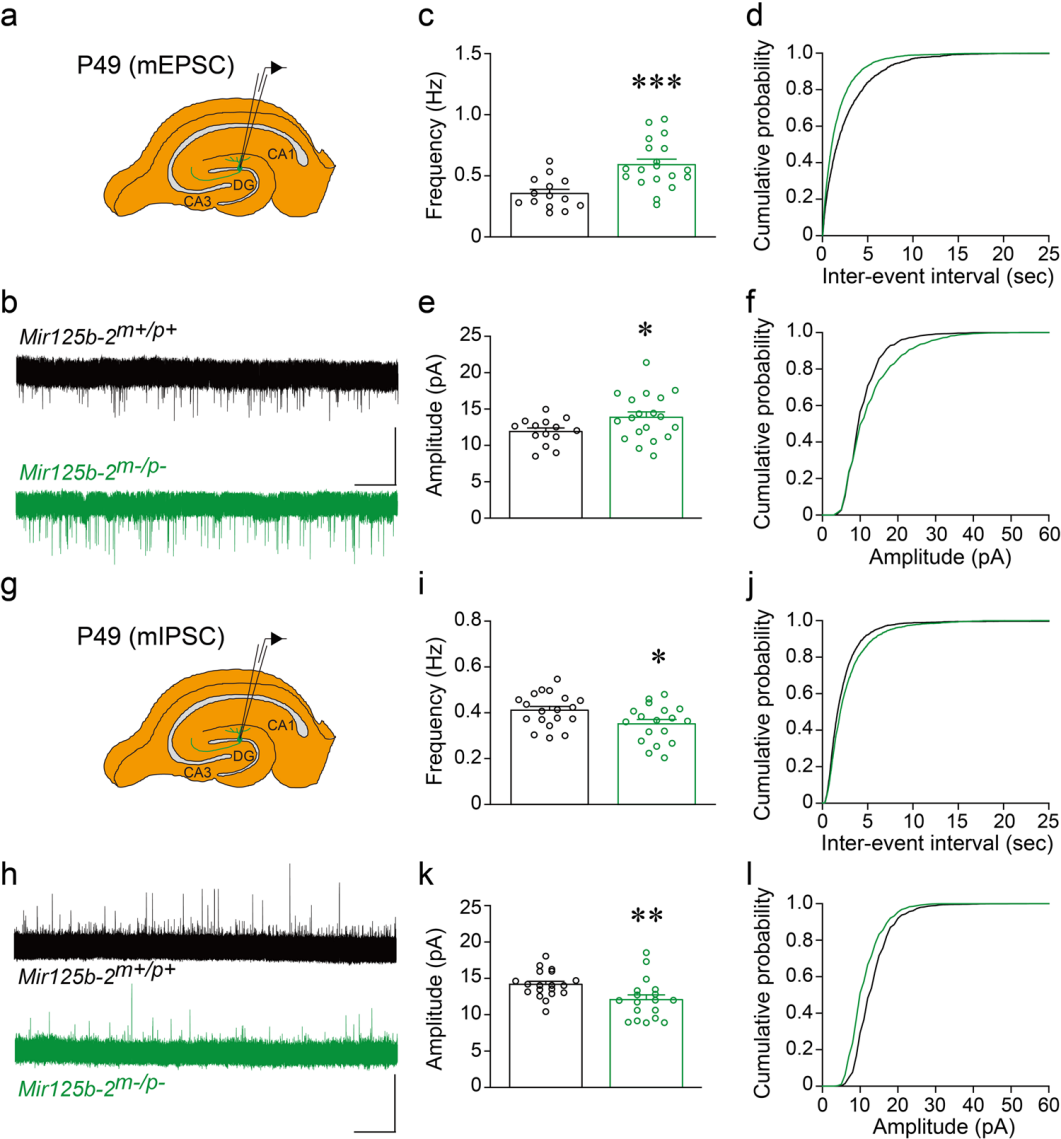
Hippocampal granule cells of *Mir125b-2*^*m*^^−^^*/p*^^−^ mice displayed impaired excitatory and inhibitory synaptic transmission. Schematic of cell patching on adult hippocampal granule cells (GC) at postnatal day 49 (P49) for measurements of miniature excitatory postsynaptic current (mEPSC) (**a**) and miniature inhibitory postsynaptic current (mIPSC) (**g**). Representative traces of mEPSC (**b**) and mIPSC (**h**) recorded from wild-type (*Mir125b-2*^*m+/p+*^) and *Mir125b-2*^*m*^^−^^*/p*^^−^ mice. Scale bar represents 20 pA and 30 s. Frequency (**c**) and cumulative probability of inter-event interval (**d**) are shown for mEPSC. Amplitude (**e**) and its cumulative probability (**f**) are shown for mEPSC. Frequency (**i**) and cumulative probability of inter-event interval (**j**) are shown for mIPSC. Amplitude (**k**) and its cumulative probability (**l**) are shown for mIPSC. mEPSC: *Mir125b-2*^*m+/p+*^, 14 cells, 3 mice; *Mir125b-2*^*m*^^−^^*/p*^^−^, 19 cells, 4 mice. mIPSC: *Mir125b-2*^*m+/p+*^, 19 cells, 4 mice; *Mir125b-2*^*m*^^−^^*/p*^^−^, 18 cells, 4 mice. Student’s *t*-test, two-tailed, *P < 0.05, **P < 0.01, ***P < 0.001. All data are presented as mean ± s.e.m.

To investigate synaptic mechanisms of deficits in hippocampus-related behaviors in *Mir125b-2*^*m*^^−^^*/p*^^−^ mice (Fig. 3), we first analyzed miniature excitatory postsynaptic currents (mEPSCs) in the hippocampal granule cells of *Mir125b-2*^*m*^^−^^*/p*^^−^ mice and their controls (*Mir125b-2*^*m+/p+*^) (Fig. 5a, b). Compared with controls, the hippocampal granule cells of *Mir125b-2*^*m*^^−^^*/p*^^−^ mice displayed increased frequency (Fig. 5c, d) and amplitude (Fig. 5e, f) of mEPSCs. When miniature inhibitory postsynaptic currents (mIPSCs) in the hippocampal granule cells of *Mir125b-2*^*m*^^−^^*/p*^^−^ mice were examined compared with their controls (Fig. 5g, h), we found decreased frequency (Fig. 5i, j) and amplitude (Fig. 5k, l) of mIPSCs. Collectively, our results suggest that miR-125b-2 regulates both excitatory and inhibitory synaptic transmission in hippocampal granule cells.

### Hippocampal expression of *Grin2a*, a synaptic molecule, was upregulated in *Mir125b-2*^*m*^^−^^*/p*^^−^ mice

**Fig. 6:**
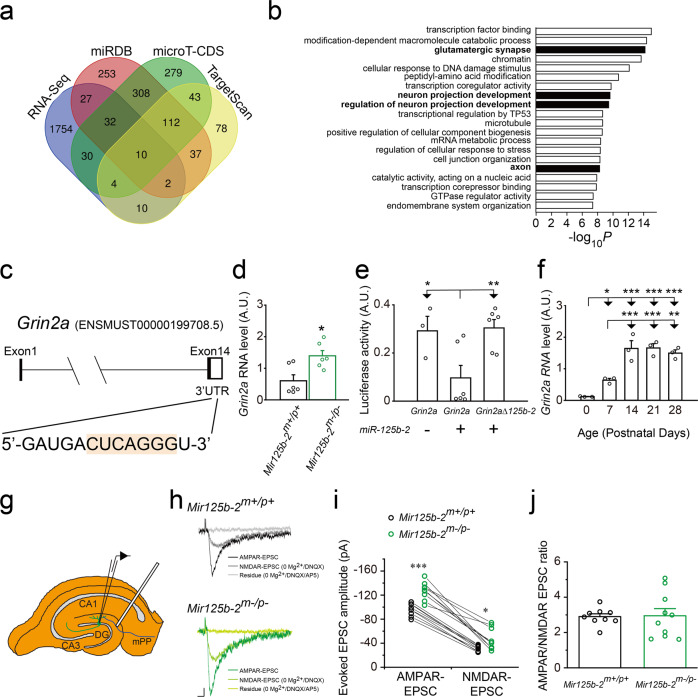
*Grin2a* was upregulated in the hippocampus of *Mir125b-2*^*m*^^−^^*/p*^^−^ mice. **a** Venn diagram showing the overlap among four databases from RNA-Seq from *Mir125b-2*^*m*^^−^^*/p+*^ mice and three software programs (miRDB, microT-CDS, and TargetScan) for predicting targets of *Mir125b-2*. **b** Gene ontology (GO) analysis of *Mir125b-2* target genes. **c** *Mir125b-2* target site on the 3′ untranslated region (3′UTR) of *Grin2a*^21^. **d** Levels of *Grin2a* in *Mir125b-2*^*m*^^−^^*/p*^^−^ mice and corresponding wild-type (*Mir125b-2*^*m+/p+*^) controls. Mann–Whitney rank-sum test. *Mir125b-2*^*m+/p+*^, *n* = 6 mice. *Mir125b-2*^*m*^^−^^*/p*^^−^, *n* = 6 mice. **e** The luciferase assay for *Grin2a* 3′UTR with and without the miR-125b-2 binding site. The luminescence ratio was calculated as the firefly luciferase signal/*Renilla* luciferase signal for each well. The *Grin2a*_mut construct was derived from *Grin2a* 3′UTR with the deletion of the miR-125b-2 binding site. **f** Levels of *Grin2a* during brain development from postnatal day 0 (P0) to P28. One-way ANOVA with Fisher LSD Method *post hoc* comparison. *n* = 3/group. *P < 0.05, **P < 0.01, *P < 0.001. **g** Schematic of cell patching on hippocampal granule cells (GC) at postnatal day 28 (P28) for measurements of evoked excitatory postsynaptic currents. **h** Representative traces of AMPAR-mediated currents (AMPAR-EPSC) and NMDAR-mediated currents (AMDAR-EPSC) recordings from granule cells of *Mir125b-2*^*m+/p+*^ (upper, black) and *Mir125b-2*^*m*^^−^^*/p*^^−^ (lower, green) mice. Scale bar represents 15 pA and 25 ms. **i** Both AMPAR- and NMDAR-mediated currents increased in *Mir125b-2*^*m*^^−^^*/p*^^−^ (green) mice compared with *Mir125b-2*^*m+/p+*^ (black) mice. **j** the ratio of amplitudes from AMPAR-mediated and NMDAR-mediated currents did not differ between *Mir125b-2*^*m+/p+*^ (black) and *Mir125b-2*^*m*^^−^^*/p*^^−^ (green) mice. Nine cells from three *Mir125b-2*^*m+/p+*^ mice; 10 cells from three *Mir125b-2*^*m*^^−^^*/p*^^−^ mice. Student’s *t*-test, two-tailed, *P < 0.05, ***P < 0.01. All data are the mean ± s.e.m.

To investigate molecular mechanisms of deficits in learning and memory and anxiety from *Mir125b-2*^*m*^^−^^*/p*^^−^ mice, we first identified potential downstream target genes of miR-125b-2 using three different bioinformatic tools (miRDB, microT-CDS, and TargetScan) in conjunction with RNA-Seq analysis results of brains from *Mir125b-2*^*m*^^−^^*/p+*^ mice at postnatal day 0 (Fig. 6a and Supplementary Data 1 and 2). We subsequently performed Gene Ontology (GO) analysis on all upregulated candidate genes from our RNA-Seq data as well as putative miR-125b-2 binding genes analyzed from miRDB, microT-CDS, and TargetScan. Our GO analysis demonstrated the candidate genes of miR-125b-2 were involved in the development of glutamatergic synapses, axons, and neuron projections (Fig. 6b). GO analysis ranked the glutamatergic synapse as highly relevant, and our RNA-Seq dataset showed three upregulated glutamate receptors: *Grin2a*, *Grik2*, and *Grm8*. Therefore, we first focused on examining expression levels of glutamate ionotropic receptor NMDA type subunit 2A (*Grin2a*) in the hippocampus of *Mir125b-2*^*m*^^−^^*/p*^^−^ mice because it is a key regulator in the glutamatergic synapse and synaptic plasticity. In general, miRNAs typically repress gene expression by binding to the 3′UTR of their target mRNAs, leading to the degradation of the mRNA. Indeed, miR-125b targeted the 3′ untranslated region (3′UTR) of *Grin2a* (Fig. 6c)^21^, and hippocampal expression of *Grin2a* in *Mir125b-2*^*m*^^−^^*/p*^^−^ mice increased compared with their controls (Fig. 6d). We did not observe any difference in levels of mRNA for *Grin1* and *Grin2b* in the hippocampus of *Mir125b-2*^*m*^^−^^*/p*^^−^ mice compared with their controls (Supplementary Fig. 5a, b). To further examine whether miR-125b-2 physically binds to the *Grin2a* 3′UTR regions and causes RNA instability, we performed luciferase assays by transfecting luciferase constructs into HEK293T cells with *Grin2a* 3′UTR or *Grin2a* 3′UTR without the miR-125b-2 binding site. The results demonstrated that overexpression of the miR-125b-2 construct resulted in a significantly higher relative luminescence ratio for *Grin2a* 3′UTR without the miR-125b-2 binding site compared with *Grin2a* 3′UTR, which was comparable to the positive control without transfection of the miR-125b-2 construct (Fig. 6e). The luciferase assay indicated the level of miR-125b-2 physically binding to *Grin2a* 3′UTR was responsible for the reduction in *Grin2a* mRNA levels. Moreover, expression of *Grin2a* was upregulated during the developmental stages of the mouse brain from P0 to P28 (Fig. 6f) and the human brain^27^. This pattern was opposite the decreased expression detected for *Mir125b-2* (Fig. 2c).

Next, to examine the functional consequences of increased *Grin2a* expression levels, we measured AMPA receptor (AMPAR)- and NMDA receptor (NMDAR)-mediated excitatory postsynaptic currents (EPSCs) in the same cells of the hippocampus from *Mir125b-2*^*m*^^−^^*/p*^^−^ mice by holding the membrane potential at −70 mV followed by electrode stimulus and sequentially adding the AMPA receptor antagonist (DNQX) and the NMDA receptor antagonist (APV) (Fig. 6g). The results showed that the peak amplitude of both AMPAR- and NMDAR-mediated EPSC increased significantly (Fig. 6h, i). However, there was no difference in the ratio of AMPA/NMDA-mediated currents (Fig. 6j).

Cholinergic neurons are densely innervated in the hippocampus and mediate hippocampus-related functions^28^. Acetylcholinesterase (ACHE) is the key enzyme for the breakdown of acetylcholine, and its 3′UTR was predicted to be targeted by miR-125b in silico^29^. However, no difference in levels of *Ache* mRNA was observed in the hippocampus of *Mir125b-2*^*m*^^−^^*/p*^^−^ mice compared with their controls using qPCR analysis (Supplementary Fig. 5c). To take full use of our RNA-Seq dataset, we comprehensively examined gene expression levels from sodium channels, potassium channels, glutamate receptors, GABA receptors, and key presynaptic molecules, which are related to the electrophysiological phenotypes we observed in *Mir125b-2*^*m*^^−^^*/p*^^−^ mice. We found several miR-125b-2 target candidates: sodium channel: *Scn8a*, and *Scn1b*; potassium channel: *Kcna4*, *Kcnc3*, *Kcnh7*, *Kcnq1*, and *Kcnq2*; glutamate receptor: *Grik2*, *Grin2a*, and *Grim8*; GABA receptor, *Gabra1*, *Gabrb2*, and *Gabbr1*; and presynaptic molecules: *Syt3*, *Syt16*, *Syt17*, *Stx3*, *Stx16*, and *Vamp4*. Details of the miR-125b-2 target candidates are summarized in Supplementary Data 3. Collectively, our results indicate miR-125b-2 binds to *Grin2a* and affects its expression in vivo.

## Discussion

Few imprinted miRNAs have been identified in the brain, and their functional roles have not been well-investigated. Our study detected an imprinted microRNA, *MIR125B2*, in human but not mouse brain. In addition, we demonstrated the critical roles of miR-125b-2 in hippocampal function and circuit. Specifically, miR-125b-2 regulated learning and memory, and anxiety. Additionally, miR-125b-2 regulates *Grin2a* expression in the hippocampal region, which affects neuronal excitability and synaptic transmission. Our findings demonstrated the complexity of imprinting patterns in different species, extended the functional roles of microRNAs in hippocampal function and circuit, and identified the downstream target of a miRNA that is important in glutamatergic synapses. Our study provides insights into miRNAs in the context of brain development and may deepen our understanding of the evolution, function, and disorders of the brain.

The species-specific status of genomic imprinting could be due to the distinct genomic elements between species. For instance, *Commd1* is imprinted in mouse but not human brain. That is because *U2af1-rs1*, an imprinted retrogene, lies within an intron of mouse *Commd1*, but the orthologous human locus lacks such an imprinted retrogene^30–32^. Our findings provide an additional example of a species-specific imprinted gene in that *MIR125B2* was imprinted in our human brain samples but not in mouse brain. It would be of future interest to identify species-specific genomic elements, differentially methylated CpG islands, or non-coding RNAs in the genomic locus of *MIR125B2* for mechanistic insights into human-specific imprinting of *MIR125B2*.

Increased hippocampal expression of *Grin2a* could partially explain the increased excitatory synaptic transmission in the hippocampal granule cells from *Mir125b-2*^*m*^^−^^*/p*^^−^ mice. The dysregulation of hippocampal function and circuit may have resulted from the collective impacts of increased evoked-postsynaptic currents, neuronal excitability, and imbalance of excitatory/inhibitory synaptic transmission. Therefore, impaired hippocampus-related behaviors in learning and memory and anxiety were observed in *Mir125b-2*^*m*^^−^^*/p*^^−^ mice. Presynaptic effects may explain changes in recorded event frequency. An optogenetic approach could be used to study presynaptic release properties using *Mir125b-2*^*m*^^−^^*/p*^^−^ mice. Identifying other miR-125b-2 targets is needed to further delineate molecular mechanisms involved in the increased neuronal excitability of hippocampal granule cells.

Down syndrome (DS) is a genetic disorder caused by triple copies of chromosome 21^33^. DS is characterized by cognitive impairment and brain hypotrophy^34^. The location of *MIR125B2* within human chromosome 21 predicts that persons with DS should have increased levels of miR-125b-2. Indeed, human studies have shown an increase in levels of miR-125b-2 in individuals with DS^35^. Our study demonstrated the important role of miR-125b-2 in hippocampus-related function and circuit, which could provide partial support for how the genetic dosage of miR-125b-2 might influence cognitive function.

Aberrant expression of miR-125b-2 has been identified in persons with cognition-related dysfunction^22–25^. Cognition-related brain regions are primarily the hippocampus and neocortex^36–38^. In this study, we established miR-125b-2 as a critical regulator in hippocampal function and circuit. It would be of interest to investigate the roles of miR-125b-2 in the neocortex in future studies.

*Mir125b-2*^*m*^^−^^*/p*^^−^ mice have deficits of short-term memory but intact long-term memory, which seems counter-intuitive. However, short-term memory and long-term memory are stored in different areas of the brain, which has been demonstrated by human studies showing that individuals can have impaired short-term memory in concert with intact long-term memory^39^. It would be of future interest to examine whether *Mir125b-2*^*m*^^−^^*/p*^^−^ mice have normal long-term memory in another behavioral paradigm.

The Y-maze rewarded alternation test used to assess the cognitive ability of rodents had been shown to be highly sensitive to hippocampal dysfunction^40^. Other brain regions, such as the cerebellum, thalamus, and substantia innominata are also involved^40^. The reduced rewarded alternation rate observed in the *Mir125b-2*^*m*^^−^^*/p*^^−^ mice indicated the presence of hippocampal dysfunction, which is also consistent with the dysfunction in excitability and synaptic transmission observed in the hippocampal granule cells.

Three widely used behavioral tests for anxiety are the open field test, light-dark box test, and elevated plus maze test. In this study, we used light as a source of anxiety in all three tests. However, the elevated plus maze test also includes height as a source of anxiety. Therefore, the discrepancy in the results of the elevated plus maze test compared with the open field test and light-dark box test in *Mir125b-2*^*m*^^−^^*/p*^^−^ mice could partially be explained by the difference in two compared with one source of anxiety.

In addition to *Grin2a*, we identified several miR-125b-2 target candidates related to neuronal excitability and synaptic transmission from our RNA-Seq dataset (Supplementary Data 3). It would be of future interest to further validate their expression levels and physical interactions with miR-125b-2. Validation of these target candidates would provide a more comprehensive picture regarding the mechanistic roles of miR-125b-2 in hippocampal neuronal and synaptic functions.

Genomic imprinting affects gene dosage, which consequently influences the phenotypes of related genes^41,42^. Our findings demonstrated that *MIR125B2* was only imprinted in the human brain, not in the mouse. As a result, the human brain has half the level of miR-125b-2 compared with mouse. Conversely, the human brain would have greater expression levels of miR-125b-2 target genes compared with their mouse counterparts. Functionally, this could make the human brain more susceptible to increased neuronal excitability and synaptic transmission compared with the mouse brain.

NMDA receptors in the brain of prenatal and neonatal rats contain predominantly GRIN1/GRIN2B; in the adult rat, there is a shift in predominance to GRIN1/GRIN2A^43^. The change in subunit composition from GRIN2B to GRIN2A influences the kinetics of excitatory postsynaptic currents, the affinity of the binding sites, and synaptic plasticity^43^. We observed increased RNA expression of *Grin2a* but not *Grin2b* and *Grin1* in the hippocampus of *Mir125b-2*^*m*^^−^^*/p*^^−^ mice compared with their controls. It would be of future interest to investigate whether increased expression of *Grin2a* contributes to a more rapid developmental switch between GRIN2B and GRIN2A in the hippocampus of *Mir125b-2*^*m*^^−^^*/p*^^−^ mice.

Although *Mir125b-2*^*m*^^−^^*/p*^^−^ mice displayed normal rates of moving, motor coordination, and balance, they showed hypoactivity. Identification of affected brain regions could provide a foundation for investigating new research lines of miR-125b-2 in brain development and function. It would also be of interest to explore molecular mechanisms of hypoactivity by identifying other targets of miR-125b-2.

Our work demonstrated the functional roles of a human-specific imprinted and brain-enriched microRNA at the molecular, neuronal, circuit, and behavioral levels, as well as its implications for the etiology of related brain disorders. Our study also opens up several new lines of research, such as species-specific imprinting, new therapeutic targeting for hippocampus-related disorders, and functional differences in isoforms with a focus on microRNA.

There are several limitations of our study. First, we did not provide the cellular resolution of the functional roles of miR-125b-2 in the hippocampus, which requires mating mice with a cell-type-specific *Cre* driver with *Mir125b-2*^*loxp/loxp*^ mice. Such information would further strengthen the mechanistic roles of miR-125b-2 in the hippocampus. Second, confirmation that GRIN2A is required for the phenotypes we observed in the hippocampus of *Mir125b-2*^*m*^^−^^*/p*^^−^ mice will require rescue experiments for those phenotypes to be conducted, which are planned for future studies. Finally, we did not explore the contribution of other learning/memory and anxiety-related brain regions in *Mir125b-2*^*m*^^−^^*/p*^^−^ mice, which will be required for confirmation that the hippocampus is the most significant brain region contributing to dysfunctional learning/memory and anxiety in *Mir125b-2*^*m*^^−^^*/p*^^−^ mice.

## Methods

### Imprinting status of *MIR125B2* in human tissue

Human tissues examined were based on availability. To identify the imprinting status of *MIR125B2* in human tissue obtained through surgical resection or postmortem, we studied one family trio, one family quartet, and one family duet. The family trio was comprised of the parents and surgical resection tissue from their 17-year-old daughter. The family quartet was comprised of the parents and postmortem tissue from two children, who were sisters, aged 29 and 32 years^5^. Samples from the family duet were comprised of a mother and surgical resection tissue from her daughter, aged 45 years. In addition, an additional family quartet was studied to identify the imprinting status of *MIR125B2* in hiPSCs and hiPSCs-derived cortical neurons. The family quartet was comprised of the parents and two offspring aged 16 years.

Surgical resection samples from the 17-year-old brain included normal and tumor tissues from the temporal cortex and parental blood. Postmortem tissue from the prefrontal cortex (Brodmann area 8) of the 29-year-old and postmortem tissue from the prefrontal cortex (Brodmann area 10) of the 32-year-old and parental blood was assessed from the family quartet. We examined surgical resection samples of normal and tumor tissues from the adrenal gland of the 45-year-old and the mother’s blood of the family duet. The assessed hiPSC-derived cortical neurons were generated using blood samples from the offspring and parents in the family quartet. The hiPSC-derived cortical neurons were collected for allele-specific expression analysis at differentiation day 35. Details of the preparation of hiPSC and hiPSCs-derived cortical neurons were mentioned previously^7^. More detailed information on the families and samples used are shown in Supplementary Fig. 1, Supplementary Data 4, and previous studies^5,7^.

The institutional review boards (IRBs) of the participating institutions approved all experimental protocols. All experiments were carried out in accordance with the approved guidelines of the IRBs of the participating institutions. Written informed consent was obtained from both parents. Human quartet samples were obtained from the University of Utah Autism Research Program. The IRBs of the University of Utah, Icahn School of Medicine, and National Taiwan University approved the analyses of samples and data in this study. Human quartet, trio, and duet samples were obtained from National Taiwan University Hospital. The IRBs of the National Taiwan University approved the analyses of samples and data in this study (IRB numbers: 201308050RINB for the human tissue study and 201901027RIND for the hiPSC study). All postmortem and surgical resection tissue was fresh-frozen, except for temporal cortex tissue from the 17-year-old, which was formalin-fixed and paraffin-embedded. All tissues were stored at −80 °C.

### SNP site selection

SNP sites used in the identification of the imprinting status of *MIR125B2* were identified by searching genomic DNAs of the offspring. Blood genomic DNA was extracted using NucleoSpin® Blood kit (Macherey-Nagel, 740951) or extracted from fresh-frozen brain tissue using NucleoSpin® Tissue kit (Macherey-Nagel, 740952). Sequences of *MIR125B2* were then amplified with specific primers (primer sequences are listed in Supplementary Data 5) and Taq polymerase (GoTaq® Master Mixes (Promega, M7122); or AptaTaq™ Fast DNA Polymerase (Roche, 6879110001) for some of the samples), in C1000 Touch Thermal Cycler (Bio-Rad Laboratories). The PCR product was purified, and Sanger sequencing was performed at Genomics Biosci & Tech company. SNP sites were chosen to be used for the identification of *MIR125B2* imprinting status if parents and offspring had heterozygous alleles, and each allele’s parent of origin could be inferred from Sanger sequencing results. Details of these SNP sites are shown in Supplementary Data 6 and Fig. 1.

### Mice

*Mir125b-2* knockout mice on a C57BL/6J background were generated (Fig. 2e). Mice were group-housed in ventilated cages on a 12-h light/dark cycle (lights off at 8 pm) with *ad libitum* access to food (PicoLab^®^ Rodent Diet 20, 5053) and water. Male *Mir125b-2* heterozygous knockout mice (HET) were mated to female C57/BL6J mice to obtain *Mir125b-2*^*m+/p*^^−^ mice. Female *Mir125b-2* HET were mated to male C57/BL6J mice to obtain *Mir125b-2*^*m*^^−^^*/p+*^ mice. Male *Mir125b-2* HET were mated to female *Mir125b-2* HET to obtain *Mir125b-2*^*m*^^−^^*/p*^^−^ mice.

The National Taiwan University College of Medicine and the College of Public Health Institutional Animal Care and Use Committee (IACUC) approved all procedures (IACUC no.: 20200084). All experiments were performed in accordance with the approved guidelines.

### Reverse transcription-quantitative PCR (RT-qPCR) analysis

Total RNA was extracted from mouse tissues using RNeasy® Lipid Tissue Mini Kit (QIAGEN, 74804) or miRNeasy Mini Kit (QIAGEN, 217004). RNAs were treated by RNase-Free DNase (QIAGEN, 79254), then reverse-transcribed by SuperScript™ III Reverse Transcriptase kit (Invitrogen, 18080044) into cDNA. Prior to qPCR analysis, samples were checked for the presence of genomic DNA. qPCR was conducted with a StepOnePlus Real-Time PCR System (Applied Biosystems) with qPCRBIO SyGreen Mix (PCR Biosystems, PB20.12-05). Samples were run in triplicate, and relative expression values were determined by the comparative Ct method (2^−ΔΔCt^). *B2m* and *Gapdh* were used as reference genes for the mouse tissues. Primer information was seen in Supplementary Data 5. For measurement of mature miR-125b-2, total RNA was added poly(A) tailing by A-Plus Poly(A) Polymerase Tailing Kit (CELLSCRIPT, C-PAP5104H), then reverse-transcribed by SuperScript^™^ II Reverse Transcriptase kit (Invitrogen, 18064022) with poly(T) adapter to generate cDNA. *5S rRNA* was used as reference genes for the mouse brains. miR-uni reverse long: GCG AGC ACA GAA TTA ATA CGA CTC AC. miR-125b-5p: TCC CTG AGA CCC TAA CTT GTG A. The age of mice was between 6 and 9 months.

### Behavioral measures

All behavioral experiments were performed with male mice. The male mice were comprised of mice derived from six litters of WT mice (*n* = 12) and four litters of *Mir125b-2*^*m*^^−^^*/p*^^−^ mice (*n* = 9). One to two weeks prior to behavioral tests, mice were handled 2–3 times per week, 1–2 min each day, to familiarize the mice with the experimenter and to reduce anxiety. Body weight was measured every 7 days before the tests and during the tests period and as a means of monitoring the health of the mice. Behavioral tests began when male mice were 7–8 weeks of age and were completed within 4–5 months with adequate time intervals between the individual tests for the mice to recover. All test mice were housed under the same conditions during the test period. To avoid confounding factors of stress and fatigue for mice during multiple behavioral tests, tests were conducted in order from the least to most stressful (Supplementary Data 7) with a gap of 2–10 days between each test, depending on the stress level of the test. The habituation time in the test room for mice was at least 30 min in most of the tests and at least 1 h habituation in the anxiety-related experiments. All behavioral tests were conducted during the dark cycle (9 am–7 pm); anxiety-related experiments were conducted from 12 pm–7 pm. Details of the behavioral tests have been described previously^7,8,44–46^.

### RNA-Seq and follow-up statistical analysis

Brains were dissected from *Mir125b-2*^*m*^^−^^*/p+*^ knockout mice and their wild-type counterparts at postnatal day 0, RNA was extracted, and ribosomal RNA was removed. Purified RNA was amplified and prepared for RNA sequencing. The generated sequences were filtered to obtain qualified reads. For differential expression analysis, CummeRbund was employed to perform statistical analyses of gene expression profiles. Details of the procedures have been previously described^4,5^. The RNA-Seq data discussed in this publication have been deposited in NCBI’s Gene Expression Omnibus and are accessible through GEO Series accession number GSE211881.

### Hippocampus brain slice preparation

The brain slice preparation procedure used in this study was modified according to previous hippocampal slice preparation and patch clamp electrophysiology procedures^46,47^ and adapted the N-methyl-D-glucamine (NMDG) protective recovery method, which provides enhanced viability of brain slices^48^. Briefly, mice were deeply anesthetized with 5% isoflurane and immediately sacrificed. The brain was then quickly removed, placed in a microtome (Leica VT1200 S, Leica), and sliced in the coronal plane at a thickness of 250 μm with ice-cold modified NMDG-HEPES dissection buffer containing (in mM): 92 NMDG, 2.5 KCl, 20 HEPES, 1.2 NaH_2_PO_4_, 30 NaHCO_3_, 10 MgSO_4_, 0.5 CaCl_2_, 3 Na-pyruvate, 2 Thiourea, 5 Na-ascorbate, and 26 D-glucose (pH 7.3; 300 mOsmol/kg). Slice recovery was performed at 32 °C for 30 min in artificial cerebrospinal fluid (ACSF), which constantly bubbled with 95% O_2_-5% CO_2_, containing (in mM): 125 NaCl, 2.5 KCl, 2 CaCl_2_, 2 MgSO_4_, 1.25 NaH_2_PO_4_, 26 NaHCO_3_, and 26 D-glucose (pH 7.3; 295~300 mOsmol/kg). After 30 min incubation at 32 °C, the incubation chamber was kept at room temperature (22–25 °C) until recording. The age of mice is around P49 for mEPSC, mIPSC, and AP measurement. The age of mice is around P28 for AMPAR- and NMDAR-mediated currents measurement.

### Electrophysiology

Individual slices were transferred to an immersion-type recording chamber mounted on an upright microscope (Axio Examiner D1, Zeiss) and continuously perfused with oxygenated ACSF at a rate of 2–3 ml/min and maintained at 32 °C. Granule cells were identified visually within the dentate gyrus through differential interference contrast (DIC) using a charge-coupled device (CCD) camera, according to cell morphology like in previous studies^46,47^. Whole-cell patch-clamp recordings were done in either current- or voltage-clamp mode using borosilicate micropipettes with internal filament (inner diameter: 0.86 mm, outer diameter: 1.5 mm, length: 10 cm; Sutter Instrument, USA). The micropipettes were pulled using a laser-based programmable microelectrode puller (P-2000, Sutter Instrument, USA), with a resistance of approximately 5–7 MΩ when filled with the internal solution. For current-clamp recording, the internal solution consisted of (in mM) 115 K-gluconate, 20 KCl, 8 NaCl, 10 HEPES, 2 Mg-ATP, 0.3 Na_2_-GTP, and 0.2 EGTA. Osmolality was set to 285–290 (mOsm) and pH to 7.3. For voltage-clamp recording, the internal solution consisted of (in mM): 140 CsCH_3_SO_3_, 2 CsCl, 5 BAPTA-TetraCs, 10 HEPES, 0.2 EGTA, 2 ATP-Mg, 0.3 GTP-Na, and 5 QX-314∙Cl (L1663, Sigma). Recordings were performed with a Multiclamp 700B (Molecular Devices) amplifier, digitized at 10 kHz, and low-pass filtered at 2 kHz with a Digidata 1440 and pClamp10 software (Molecular Devices). Data were analyzed offline by pClamp 10.7 acquisition software (Molecular Devices, USA). Only cell access resistance lower than 10 MΩ was accepted, and recordings with variations greater than 15% of the baseline resistance value were excluded.

The properties of a single AP were monitored from the first AP elicited by the step depolarizing protocol. The AP spike was measured from the threshold to the peak of the spike. The AP threshold was determined when dVm/dt reached close to 10 V/s. The half-width of the AP was measured as the width at the half-maximal spike. The fast and the medium after-hyperpolarization were determined as the voltage difference between the threshold and the early and the late negative voltage point, respectively, after the AP spike. The membrane time constant was measured from the voltage response between 0 pA and a 0.5-s hyperpolarizing current injection of −30 pA. To elucidate the mechanisms of spontaneous transmitter release at different synapses, we recorded both inhibitory postsynaptic currents (mIPSCs) and miniature excitatory postsynaptic currents (mEPSCs) in dentate gyrus granule cells under standard conditions of pharmacological isolation. The mEPSCs were isolated with the addition of 1 μM TTX (ab120055, Abcam), 5 μM SR95531 (ab120042, Abcam), and 1 μM strychnine (ab120416, Abcam) into the ACSF. The mIPSCs were isolated with 1 μM TTX, 5 mM kynurenic acid (ab120064, Abcam), 1 μM strychnine, and voltage-clamped at 0 mV. Both mEPSCs and mIPSCs were measured and analyzed using the Mini-Analysis program (Synaptosoft Inc., Fort Lee, NJ, USA) or the Clampfit 10.4 (Molecular Devices, Sunnyvale, CA, USA).

For excitatory postsynaptic currents (EPSCs) recordings, cells were maintained at −70 mV in voltage-clamp mode with 100 μM picrotoxin (ab120315, Abcam) and 1 μM strychnine (ab120416, Abcam) supplemented ACSF. The internal solution consisted of (mM): 100 CsCH_3_SO_3_, 15 CsCl, 2.5 MgCl_2_, 10 HEPES, 5 QX-314∙Cl (L1663, Sigma), 5 BAPTA-TetraCs, 4 ATP-Mg, 0.3 GTP-Na, and 0.025 Alexa Fluor 594 (pH, 7.2; 295 mOsmol). A concentric bipolar stainless-steel electrode (CBCMX75 (ST1), FHC, Inc., Bowdoinham, ME, USA) was placed in the medial aspect of the molecular layer of the DG. EPSCs were evoked by supplying biphasic voltage pulses (100 μs, 0.5–1 V). For each slice, stimulus intensity was adjusted to yield 50–55% of the maximal response amplitude. Evoked currents were measured every 15 s. α-Amino-3-hydroxyl-5-methyl-isoazole-propionate (AMPA) receptor-mediated currents (AMPAR-mediate EPSCs) were determined by comparing the difference between the pre-stimulation baseline and the evoked EPSC peak. NMDA receptor-mediated currents (NMDAR-mediated EPSCs) were isolated by replacing ACSF with Mg^2+^-free ACSF in the presence of the AMPA receptor antagonist DNQX (20 μM) (ab120169, Abcam) and was further confirmed by adding 25 μM AP5 (ab120271, Abcam). Amplitudes for evoked EPSCs were analyzed using Clampfit 10.2 (Molecular Devices). NMDAR- and AMAPR-mediated currents were both measured in the same cells in the hippocampus.

### Luciferase assay

*Mir125b-2* expression construct, firefly (FF) luciferase construct, and *Renilla* (RR) luciferase construct were co-transfected with a copy number ratio of 4:2:1, respectively, into HEK293T cells by Lipofectamine™ 2000 Transfection Reagent (Invitrogen, 11668019). After transfection of 48 h, the luciferase assay was performed with the Dual-Glo® Luciferase Assay System (Promega, E2920), and luminescence was acquired with SpectraMax i3x Multi-Mode Microplate Reader (Molecular Devices). Positive controls were co-transfected with *Grin2a* 3′UTR-firefly luciferase and *Renilla* luciferase constructs. The luminescence ratio was calculated as the firefly luciferase signal/*Renilla* luciferase signal for each well.

### DNA constructs for luciferase assays

For the miR-125b-2 overexpression construct, we used the pcDNA™3.1 (+) Mammalian Expression Vector (Invitrogen, V79020) as the backbone, and pri-miR-125b-2 was driven by the short form *EF1a* promoter, rather than the *CMV* promoter. For luciferase, we used the firefly luciferase construct—pGL3-control vector (Promega, E1741)—as the backbone to insert *Grin2a* 3′UTR or *Grin2a* 3′UTR without the miR-125b-2 binding site (*Grin2a*_mut) after the firefly luciferase coding sequence. *Renilla* luciferase expression was performed with the pGL4.74[hRluc/TK] Vector (Promega, E6921). Details of the sequence are listed in Supplementary Data 8.

### Western blotting

Tissue was homogenized in 1×RIPA lysis buffer [150 mM NaCl, 50 mM Tris/HCl, 5 mM EDTA, 0.5% sodium deoxycholate, 1% Nonidet P-40, 0.1% SDS) with a protease inhibitor cocktail (Hycell, HC100-007)]. Following centrifugation, the supernatant was collected, and protein concentration was measured with the Pierce BCA Protein Assay Kit (Thermo Scientific, 23227). Total protein lysates (30 μg) were separated by 6% (stacking layer)/10% (separating layer) SDS-PAGE and transferred to nitrocellulose membranes. Immunoblotting was performed using the following primary antibodies: mouse anti-Gephyrin (1:1000, Synaptic Systems, 147111), mouse anti-Glial Fibrillary Acidic Protein (1:1000, Sigma, G3893), mouse anti-NeuN (1:1000, Merck, MAB377), rabbit anti-Neurofilament (1:1000, Abcam, ab8135), mouse PSD95 (1:2000, Thermo Fisher, 7E3-1B8), rabbit anti-Synapsin I (1:1000, Merck, AB1543), and rabbit anti-GAPDH (1:6000, Genetex, GTX110118). Primary antibodies were detected with their corresponding secondary antibodies: IRDye680 donkey anti-mouse IgG (H + L) (1:15,000, LI-COR, 926-68072) and IRDye800CW donkey anti-rabbit IgG (H + L) (1:15,000, Li-COR, 926-32213). Protein bands were visualized with an Odyssey^®^ Fc Dual-Mode Imaging System (LI-COR Biosciences). To control for protein loading, each protein level was normalized to the GAPDH level detected in each sample. The age of mice is between 6 and 9 months.

### Statistics and reproducibility

All data are presented as means ± standard error of the mean (s.e.m.) with sample sizes (*n*) shown in figures or stated in the text. Statistical analyses were performed using SigmaPlot 11 (Systat Software). Normality tests (Shapiro–Wilk) and equal variance tests were run and passed (*P* > 0.05) before parametric statistical analyses were performed. Non-parametric statistical analyses were performed if normality and equal variance tests were not passed (*P* < 0.05).

### Reporting summary

Further information on research design is available in the Nature Portfolio Reporting Summary linked to this article.

## Supplementary information

Supplementary Information

Description of Additional Supplementary Files

Supplementary Data 1

Supplementary Data 2

Supplementary Data 3

Supplementary Data 4

Supplementary Data 5

Supplementary Data 6-7

Supplementary Data 8

Supplementary Data 9-13

Reporting Summary

## Data Availability

The numerical source data for graphs are available in Supplementary Data 9–13. Uncropped gels are available in Supplementary Fig. 6. RNA-Seq data are accessible through GEO Series accession number GSE211881. All three newly generated plasmids are available at the BioMed Resource Core of the 1^st^ Core Facility Lab, NTU-CM. All other data are available from the corresponding author upon reasonable request.
