# Supplementary material for: Mir125b-2 imprinted in human but not mouse brain regulates hippocampal function and circuit in mice
Source: Commun Biol. 2023 Mar 14;6:267. doi: 10.1038/s42003-023-04655-y (PMC10014956; doi:10.1038/s42003-023-04655-y)
Supplement: Supplementary file 1 — Supplementary Information [file 42003_2023_4655_MOESM1_ESM.pdf]

Mir125b-2 imprinted in human but not mouse brain regulates hippocampal function and circuit in mice

Ming-Yi Chou[1,†], Xuhui Cao[1,†], Kuan-Chu Hou[1,†], Meng-Han Tsai[1], Chih-Yu Lee[1,2], Meng-Fai Kuo[3], Vin-Cent Wu[4], Hsin-Yi Huang[5], Schahram Akbarian[6], Sheng-Kai Chang[7], Chung-Yi Hu[7], Shu-Wha Lin[7], and Hsien-Sung Huang[1,*]

SUPPLEMENTARY MATERIAL

SUPPLEMENTARY DATA

Supplementary Data 1. Details of bioinformatic information.

Supplementary Data 2. List of up- and down-regulated genes in the RNA-Seq.

Supplementary Data 3. List of electrophysiology-related genes in the RNA-Seq.

Supplementary Data 4. Details of human subjects.

Supplementary Data 5. Details of primer information.

Supplementary Data 6. Details of *MIR125B* SNPs.

Supplementary Data 7. Schedules of mouse behavioral tests.

Supplementary Data 8. Details of DNA constructs for luciferase assays.

Supplementary Data 9. The numerical source data for graphs in figure 2.

Supplementary Data 10. The numerical source data for graphs in figure 3.

Supplementary Data 11. The numerical source data for graphs in figure 4.

Supplementary Data 12. The numerical source data for graphs in figure 5.

Supplementary Data 13. The numerical source data for graphs in figure 6.

SUPPLEMENTARY FIGURES

Supplementary Figure 1. Validation of SNP status of *MIR125B2* in human cohorts.

Supplementary Figure 2. No changes of neuronal, glial, and synaptic integrity in the hippocampus of adult *Mir125b-2*$^{m-/p-}$ mice.

Supplementary Figure 3. No changes of neuronal, glial, and synaptic integrity in the cerebral cortex of adult *Mir125b-2*$^{m-/p-}$ mice.

Supplementary Figure 4. *Mir125b-2*$^{m-/p-}$ mice showed normal motor function, fear-related learning and memory, and depressive-like behavior.

Supplementary Figure 5. No change of *Grin1*, *Grin2b*, and *Ache* mRNA levels in the hippocampus of *Mir125b-2*$^{m-/p-}$ mice.

Supplementary Figure 6. Uncropped gel images for figure 2f.

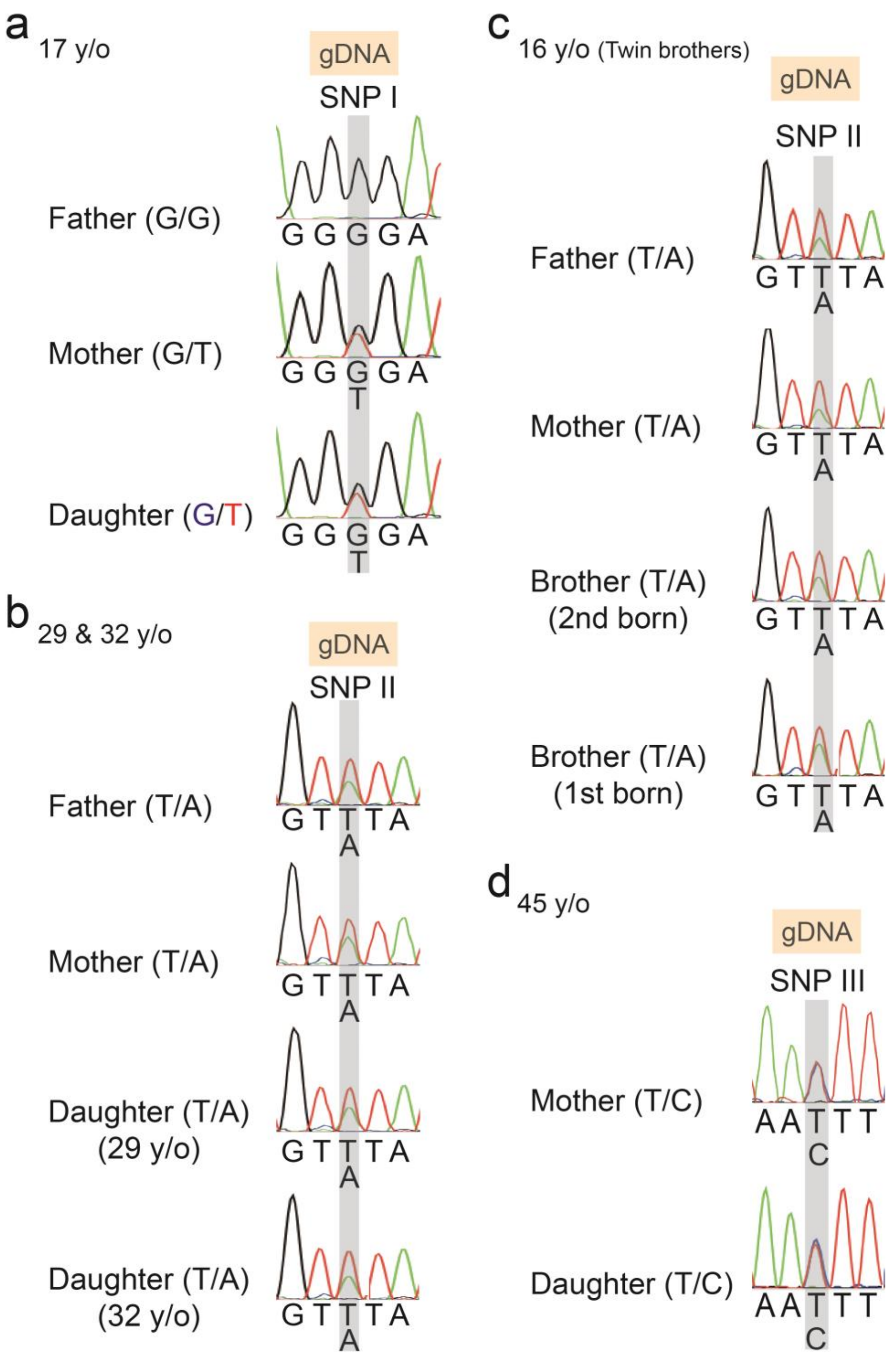

Supplementary Figure 1. Validation of SNP status near *MIR125B2* in human cohorts. SNP sites near *MIR125B2* were validated by Sanger sequencing in human cohorts (**a-d**). Single nucleotide polymorphism sites are labeled with gray columns.

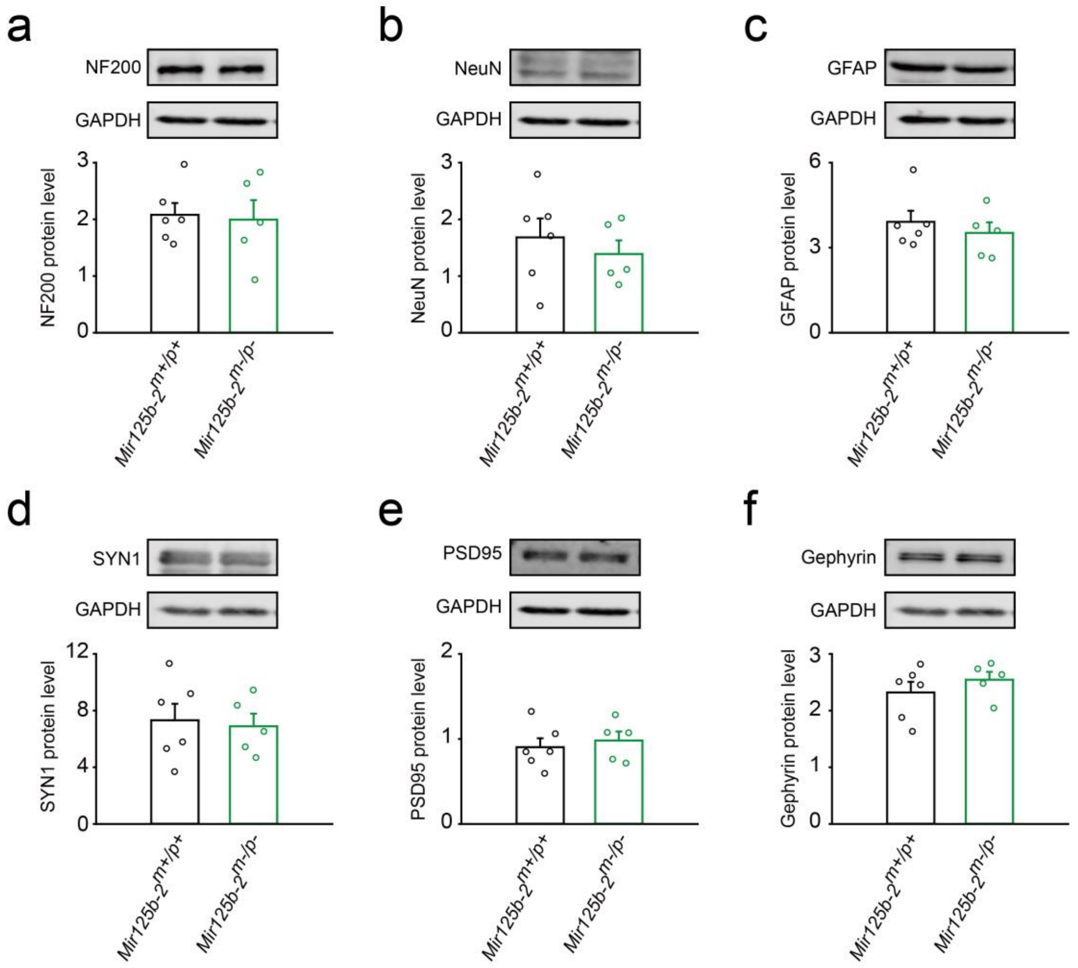

Supplementary Figure 2. No changes of neuronal, glial, and synaptic integrity in the hippocampus of adult *Mir125b-2*$^{m-/p-}$ mice. Western blot analysis was performed with hippocampal extracts from adult *Mir125b-2*$^{m-/p-}$ mice for different protein markers as follows. (**a**) NF200, (**b**) NeuN, (**c**) GFAP, (**d**) SYN1, (**e**) PSD95, and (**f**) Gephyrin. *Mir125b-2*$^{m+/p+}$, n = 6 mice; *Mir125b-2*$^{m-/p-}$, n = 5 mice. All data are the mean ± s.e.m.

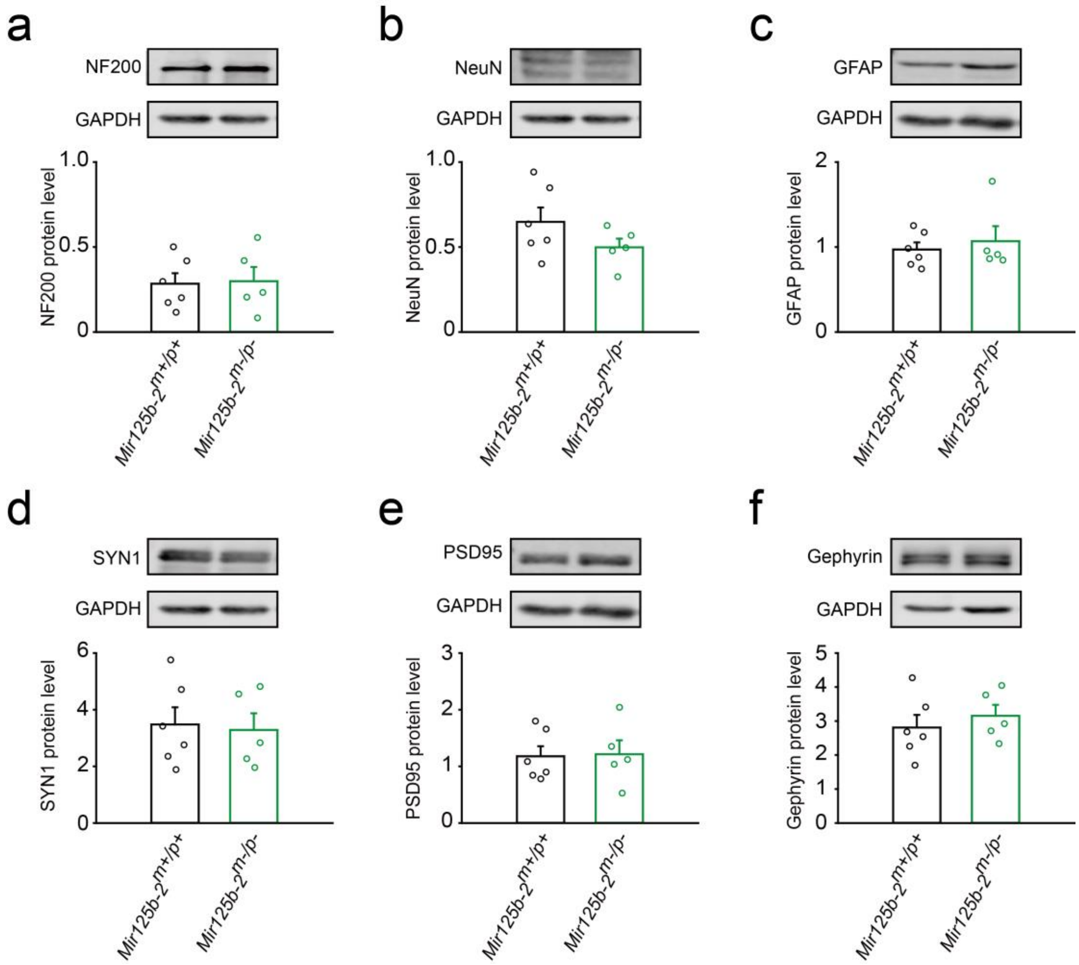

Supplementary Figure 3. No changes of neuronal, glial, and synaptic integrity in the cerebral cortex of adult *Mir125b-2*$^{m-/p-}$ mice. Western blot analysis was performed with extracts from the cerebral cortex of adult *Mir125b-2*$^{m-/p-}$ mice for different protein markers as follows. (**a**) NF200, (**b**) NeuN, (**c**) GFAP, (**d**) SYN1, (**e**) PSD95, and (**f**) Gephyrin. *Mir125b-2*$^{m+/p+}$, n = 6 mice; *Mir125b-2*$^{m-/p-}$, n = 5 mice. All data are the mean ± s.e.m.

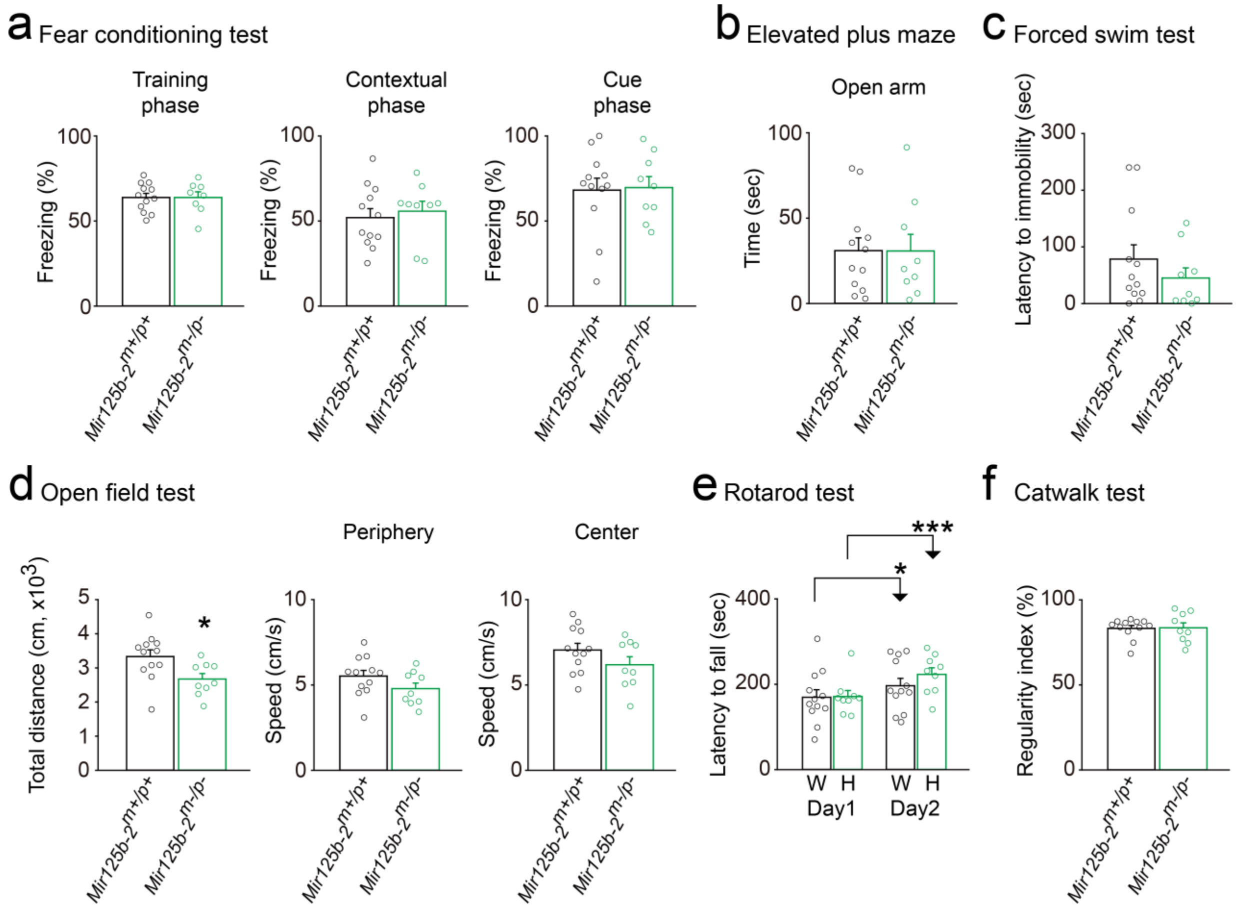

Supplementary Figure 4. *Mir125b-2$^{m-/p-}$* mice showed normal motor function, fear-related learning and memory, and depressive-like behavior. Bar graphs showing differences in behavioral tests from *Mir125b-2$^{m-/p-}$* mice and their corresponding wild-type (*Mir125b-2$^{m+/p+}$*) controls. (**a**) Percentage of time spent freezing (no movement) was measured during training (left), contextual (middle), and cue (right) phase in the fear conditioning test. (**b**) Time spent in the open arm was measured with the elevated plus maze test. (**c**) Latency to immobility was measured with the forced swim test. (**d**) Total distance (left), mean speed in the periphery (middle), and mean speed in the center were measured with the open field test. (**e**) Latency to fall was measured on day 1 and day 2 with the rotarod test. (**f**) Regularity index was measured with the catwalk test. Two-way repeated measures ANOVA with Fisher LSD Method *post hoc*

comparison, and Student’s t test, two-tailed. *$P < 0.05$. *Mir125b-2*$^{m+/p+}$, n = 12; *Mir125b-2*$^{m-/p-}$, n = 9. All data are the mean ± s.e.m.

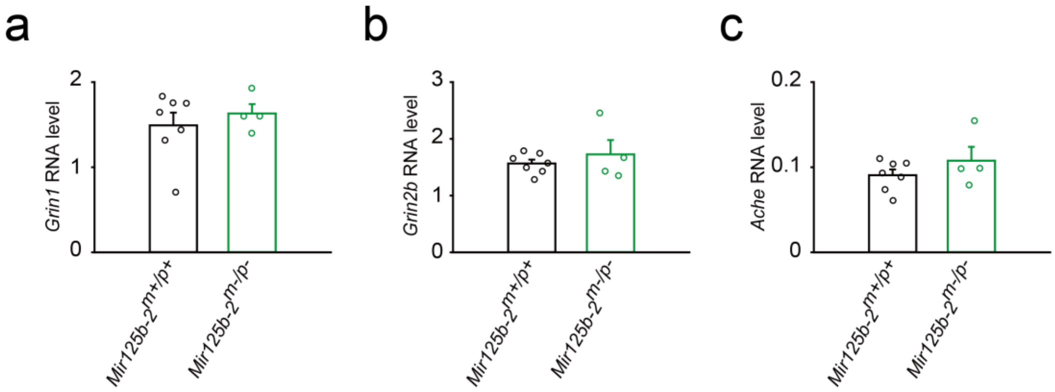

Supplementary Figure 5. No change of *Grin1*, *Grin2b*, and *Ache* mRNA levels in the hippocampus of *Mir125b-2*$^{m-/p-}$ mice. mRNA levels of *Grin2* (**a**), *Grin2b* (**b**), and *Ache* (**c**) were measured from extracts of the adult hippocampus of *Mir125b-2*$^{m-/p-}$ mice and their control mice with qPCR analysis. *Mir125b-2*$^{m+/p+}$, n = 7; *Mir125b-2*$^{m-/p-}$, n = 4. All data are the mean ± s.e.m.

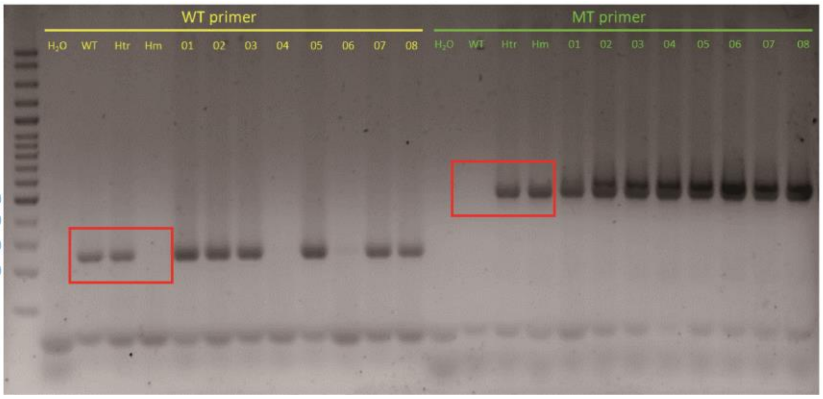

Supplementary Figure 6. Uncropped gel images for figure 2f. Red rectangle represents cropped areas.
