# Supplementary material for: Mir125b-2 imprinted in human but not mouse brain regulates hippocampal function and circuit in mice
Source: Commun Biol. 2023 Mar 14;6:267. doi: 10.1038/s42003-023-04655-y (PMC10014956; doi:10.1038/s42003-023-04655-y)
Supplement: Supplementary file 2 — Description of Additional Supplementary Files [file 42003_2023_4655_MOESM2_ESM.pdf]

Description of Additional Supplementary Files

File name: Supplementary Data 1

Description: Details of bioinformatic information.

File name: Supplementary Data 2

Description: List of up- and down-regulated genes in the RNA-Seq.

File name: Supplementary Data 3

Description: List of electrophysiology-related genes in the RNA-Seq.

File name: Supplementary Data 4

Description: Details of human subjects.

File name: Supplementary Data 5

Description: Details of primer information.

File name: Supplementary Data 6

Description: Details of MIR125B SNPs.

File name: Supplementary Data 7

Description: Schedules of mouse behavioral tests.

File name: Supplementary Data 8

Description: Details of DNA constructs for luciferase assays..

File name: Supplementary Data 9

Description: The numerical source data for graphs in figure 2.

File name: Supplementary Data 10

Description: The numerical source data for graphs in figure 3.

File name: Supplementary Data 11

Description: The numerical source data for graphs in figure 4.

File name: Supplementary Data 12

Description: The numerical source data for graphs in figure 5.

File name: Supplementary Data 13

Description: The numerical source data for graphs in figure 6.
