# Supplementary material for: Mir125b-2 imprinted in human but not mouse brain regulates hippocampal function and circuit in mice
Source: Commun Biol. 2023 Mar 14;6:267. doi: 10.1038/s42003-023-04655-y (PMC10014956; doi:10.1038/s42003-023-04655-y)
Supplement: Supplementary file 11 — Reporting Summary [file 42003_2023_4655_MOESM11_ESM.pdf]

nature portfolio

Corresponding author(s): Hsien-Sung Huang

Last updated by author(s): 2023/02/24

Reporting Summary

Nature Portfolio wishes to improve the reproducibility of the work that we publish. This form provides structure for consistency and transparency in reporting. For further information on Nature Portfolio policies, see our Editorial Policies and the Editorial Policy Checklist.

Statistics

For all statistical analyses, confirm that the following items are present in the figure legend, table legend, main text, or Methods section.

n/a	Confirmed	
☐	☒	The exact sample size (*n*) for each experimental group/condition, given as a discrete number and unit of measurement
☐	☒	A statement on whether measurements were taken from distinct samples or whether the same sample was measured repeatedly
☐	☒	The statistical test(s) used AND whether they are one- or two-sided<br>*Only common tests should be described solely by name; describe more complex techniques in the Methods section.*
☒	☐	A description of all covariates tested
☐	☒	A description of any assumptions or corrections, such as tests of normality and adjustment for multiple comparisons
☐	☒	A full description of the statistical parameters including central tendency (e.g. means) or other basic estimates (e.g. regression coefficient) AND variation (e.g. standard deviation) or associated estimates of uncertainty (e.g. confidence intervals)
☒	☐	For null hypothesis testing, the test statistic (e.g. *F*, *t*, *r*) with confidence intervals, effect sizes, degrees of freedom and *P* value noted<br>*Give P values as exact values whenever suitable.*
☒	☐	For Bayesian analysis, information on the choice of priors and Markov chain Monte Carlo settings
☒	☐	For hierarchical and complex designs, identification of the appropriate level for tests and full reporting of outcomes
☒	☐	Estimates of effect sizes (e.g. Cohen's *d*, Pearson's *r*), indicating how they were calculated

Our web collection on statistics for biologists contains articles on many of the points above.

Software and code

Policy information about availability of computer code

Data collection	N/A
Data analysis	N/A

For manuscripts utilizing custom algorithms or software that are central to the research but not yet described in published literature, software must be made available to editors and reviewers. We strongly encourage code deposition in a community repository (e.g. GitHub). See the Nature Portfolio guidelines for submitting code & software for further information.

Data

Policy information about availability of data

All manuscripts must include a data availability statement. This statement should provide the following information, where applicable:

- Accession codes, unique identifiers, or web links for publicly available datasets
- A description of any restrictions on data availability
- For clinical datasets or third party data, please ensure that the statement adheres to our policy

The numerical source data for graphs are available in Supplementary Data 9-13. Uncropped gels are available in Supplementary Figure 6. RNA-Seq data are accessible through GEO Series accession number GSE211881. All three newly generated plasmids are available at the BioMed Resource Core of the 1st Core Facility Lab, NTU-CM. All other data are available from the corresponding author upon reasonable request.

Human research participants

Policy information about studies involving human research participants and Sex and Gender in Research.

Reporting on sex and gender	Male and female are included.
Population characteristics	All detailed information was summarized in Supplementary Data 4.
Recruitment	Participants were recruited when they visited their doctors.
Ethics oversight	National Taiwan University Hospital

Note that full information on the approval of the study protocol must also be provided in the manuscript.

Field-specific reporting

Please select the one below that is the best fit for your research. If you are not sure, read the appropriate sections before making your selection.

☒ Life sciences ☐ Behavioural & social sciences ☐ Ecological, evolutionary & environmental sciences

For a reference copy of the document with all sections, see nature.com/documents/nr-reporting-summary-flat.pdf

Life sciences study design

All studies must disclose on these points even when the disclosure is negative.

Sample size	Sample size is sufficient for data analysis and statistical analysis.
Data exclusions	No data were excluded from analysis.
Replication	Data can be reproduced.
Randomization	Mice were grouped into wild-type and Mir125b-2 knockout.
Blinding	Investigators were blind to animal genotypes when performing experiments

Reporting for specific materials, systems and methods

We require information from authors about some types of materials, experimental systems and methods used in many studies. Here, indicate whether each material, system or method listed is relevant to your study. If you are not sure if a list item applies to your research, read the appropriate section before selecting a response.

Materials & experimental systems

n/a	Involved in the study	
☐	☒	Antibodies
☐	☒	Eukaryotic cell lines
☒	☐	Palaeontology and archaeology
☐	☒	Animals and other organisms
☒	☐	Clinical data
☒	☐	Dual use research of concern

Methods

n/a	Involved in the study	
☒	☐	ChIP-seq
☒	☐	Flow cytometry
☒	☐	MRI-based neuroimaging

Antibodies

Antibodies used	1) Primary antibodies: mouse anti-Gephyrin (1:1000, Synaptic Systems, 147111), mouse anti-Glial Fibrillary Acidic Protein (1:1000, Sigma, G3893), mouse anti-NeuN (1:1000, Merck, MAB377), rabbit anti-Neurofilament (1:1000, abcam, ab8135,), mouse PSD-95 (1:2000, Thermo Fisher, 7E3-1B8), rabbit anti-Synapsin I (1:1000, Merck, AB1543) and rabbit anti-GAPDH (1:6000, Genetex, GTX110118).<br>2) Secondary antibodies: IRDye680 donkey anti-mouse IgG (H+L) (1:15000, LI-COR, 926-68072) and IRDye800CW donkey anti-rabbit IgG (H+L) (1:15000, Li-COR, 926-32213).

Validation	N/A

Eukaryotic cell lines

Policy information about cell lines and Sex and Gender in Research

Cell line source(s)	HEK293 cells were originally isolated from the kidney of a human embryo.
Authentication	N/A
Mycoplasma contamination	No
Commonly misidentified lines (See ICLAC register)	No

Animals and other research organisms

Policy information about studies involving animals; ARRIVE guidelines recommended for reporting animal research, and Sex and Gender in Research

Laboratory animals	Adult B6 mice
Wild animals	N/A
Reporting on sex	Both male and female
Field-collected samples	N/A
Ethics oversight	National Taiwan University College of Medicine

Note that full information on the approval of the study protocol must also be provided in the manuscript.
